# Reconstruction Procedures for Osteoporotic Thoracolumbar Vertebral Fractures With Neurological Deficits: A Retrospective Comparison of the Lateral-Posterior Combined Approach and the Posterior-Only Approach With Three-Column Osteotomy

**DOI:** 10.7759/cureus.107669

**Published:** 2026-04-24

**Authors:** Yuichi Ono, Yuji Kasukawa, Daisuke Kudo, Ryota Kimura, Takashi Kobayashi, Ryo Shoji, Tetsuya Suzuki, Michio Hongo, Naohisa Miyakoshi

**Affiliations:** 1 Orthopedic Surgery, Akita University Graduate School of Medicine, Akita, JPN; 2 Rehabilitation, Akita University Hospital, Akita, JPN; 3 Orthopedic Surgery, Akita Kousei Medical Center, Akita, JPN; 4 Orthopedic Surgery, Nakadori General Hospital, Akita, JPN; 5 Physical Therapy, Akita University Graduate School of Medicine, Akita, JPN

**Keywords:** anterior column reconstruction, local kyphosis angle, neurological deficits, osteoporosis, osteoporotic vertebral fractures

## Abstract

Introduction

Surgical strategies for osteoporotic vertebral fractures (OVFs) with neurological symptoms remain controversial. Although anterior column reconstruction is recommended for severe vertebral collapse, few comparative studies have evaluated the lateral-posterior combined approach (LP approach) versus the posterior-only approach (P approach) that incorporates three-column osteotomy and cage reconstruction. This retrospective study compared perioperative, radiographic, and functional outcomes between these two approaches in patients with OVFs and neurological deficits.

Materials and methods

Thirty-eight patients (median age: 75 years; 6 men and 32 women) with thoracolumbar OVFs and neurological deficits were retrospectively analyzed. Twenty-two patients underwent the LP approach (LP group), and 16 underwent the P approach (P group), which involved vertebral column resection and cage insertion. Outcomes evaluated over a two-year follow-up included reoperation rates, neurological status, activities of daily living (ADL), intraoperative blood loss, kyphotic angle correction, and mechanical complications.

Results

There were no significant differences between the two groups in reoperation rates, neurological recovery, or ADL improvement. Intraoperative blood loss was significantly lower in the LP group (p < 0.05). Both groups achieved satisfactory correction of kyphotic deformities, although correction loss at two years was slightly greater in the LP group. Rates of perioperative and postoperative events, including implant-related complications, did not show clear between-group differences.

Conclusions

In this multicenter retrospective cohort, both the LP approach and P approach were associated with postoperative neurological and functional improvement at two years. The LP approach was associated with lower blood loss, whereas correction loss at two years was smaller in the P group.

## Introduction

Osteoporotic vertebral fractures (OVFs) are among the most common osteoporotic fragility fractures, with a reported prevalence of 18-51% in older adults, particularly women [1]. OVFs are mainly treated with conservative therapy, including rest, immobilization, analgesics, bracing, physical therapy, and osteoporotic treatment [2,3]. However, conservative treatment is sometimes unsuccessful, leading to nonunion and vertebral collapse after osteoporotic thoracolumbar compression fractures [4]. Some patients develop neurological symptoms due to retropulsion of bone fragments into the spinal canal, progressive kyphosis, instability at the fracture site, and neural compression [5]. Vertebral collapse has been reported to progress in 7-37% of patients, and a small minority (approximately 2%) ultimately develop neurological deficits [6,7]. Patients with neurological symptoms often require surgery due to reduced quality of life (QOL) and activities of daily living (ADL).

Surgical treatment of OVFs requires decompression of neural elements and restoration of spinal stability, and various surgical approaches have been reported [8-10]. However, the optimal surgical approach remains controversial, since these elderly patients have several comorbidities and frequent instrumentation failure due to bone fragility. In particular, the controversy concerns which reconstructive strategy to use (e.g., anterior, posterior, or minimally invasive lateral-posterior combined approach (LP approach)). Because direct comparative studies between these approaches remain limited, the optimal strategy and patient selection criteria remain poorly established [11].

In Japan, the posterior-only approach (P approach) has traditionally been used for OVFs with neurological deficit [12]. This includes posterior vertebral column resection or three-column osteotomy (3CO) (Schwab grade 4 or 5) with cage-based anterior column reconstruction and posterior fixation [13]. In contrast, thoracolumbar junction corpectomy and expandable cage reconstruction via a minimally invasive lateral approach (e.g., XLIF, NuVasive, San Diego, CA, USA) have been increasingly adopted as less invasive options, potentially reducing posterior soft-tissue disruption and blood loss [14-16].

Despite increasing use of the LP approach, direct comparative evidence between the LP and P approaches for OVFs with neurological deficits remains limited, particularly regarding mid-term reoperation rates, mechanical complications, radiographic correction/maintenance, and postoperative functional outcomes at two-year follow-up. Therefore, this retrospective study compared outcomes between the LP approach with X-Core (LP group) and the P approach (P group) in patients with OVFs and neurological deficits.

The primary objective was to describe and compare (1) perioperative invasiveness (operative time and blood loss), (2) radiographic correction/maintenance (local kyphosis angle), and (3) two-year clinical outcomes (reoperation, Frankel grade, and ADL), along with perioperative and postoperative events including implant-related complications and adjacent vertebral fractures. We expected that both strategies would be associated with postoperative neurological and functional improvement, while perioperative invasiveness and patterns of mechanical complications might differ between approaches.

## Materials and methods

**Figure 1:**
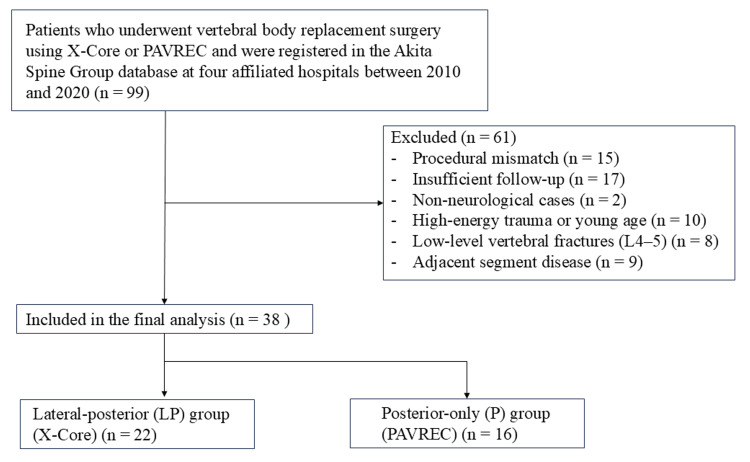
Flowchart of patient selection Thirty-eight patients were divided into two surgical procedure groups (LP group and P group). PAVREC: posterior-approach vertebral replacement with rectangular parallelepiped cages, LP group: lateral-posterior combined approach group, P group: posterior-only approach group

This was a retrospective, multicenter study conducted with institutional review board approval for medical record review. Four hospitals affiliated with the Akita Spine Group participated in the study. Patients with delayed OVFs who underwent vertebral body replacement surgery using either the LP approach with X-Core or the P approach between 2010 and 2020 were identified from the Akita Spine Group database (n = 99). After excluding patients due to procedural mismatch (procedures not fitting the predefined LP or P approach categories), insufficient follow-up, absence of neurological symptoms (cases in which the primary surgical objective was kyphosis correction without neurological deficit), high-energy trauma (because the injury mechanism and clinical course differ from osteoporotic fragility fractures), or fracture at L4-5 (because vertebral body replacement via a lateral approach can be technically challenging or not feasible at these levels, reducing comparability between groups), a total of 38 cases (6 men and 32 women) were included in the final analysis (Figure 1).

These patients were categorized into two groups: the LP group (n = 22) and the P group (n = 16). Surgeries were performed by six spine surgeons across the participating institutions. The surgeon's judgment determined the choice of approach without predefined selection criteria. No standardized decision algorithm was used to allocate patients to the LP approach or P approach.

The inclusion criteria were as follows: delayed OVF with neurological deficit after failure of initial conservative treatment; age ≥60 years; a minimum postoperative follow-up period of two years; and thoracolumbar fractures located between T10 and L3. Neurological deficit was defined as objective motor weakness and/or sensory disturbance attributable to the index OVF. Clinically significant radicular leg pain or neurogenic claudication was included only when it was consistent with radiographic neural compression on magnetic resonance imaging or computed tomography. The exclusion criteria included acute high-energy trauma, pathological fractures, absence of neurological symptoms, prior thoracolumbar surgery, and treatment with vertebroplasty or balloon kyphoplasty without intervertebral fixation. Basic demographic and surgical data (age, sex, preoperative illness duration, and postoperative follow-up period) were recorded.

This retrospective study was approved by the Institutional Review Board of Akita University Graduate School of Medicine (IRB approval no. 1879). It was performed in accordance with the ethical standards established in the Declaration of Helsinki.

Surgical procedures

Lateral-Posterior Combined Approach

**Figure 2:**
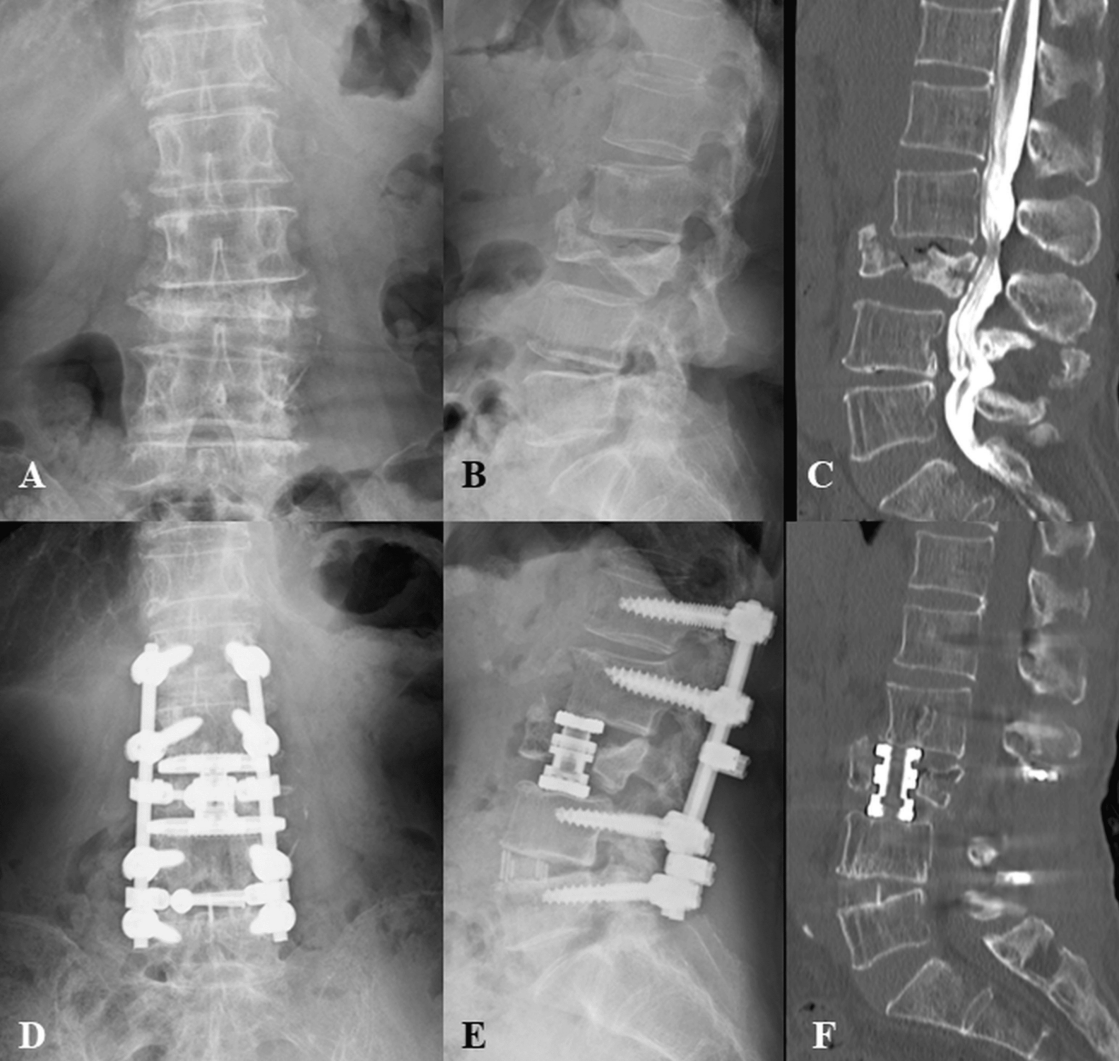
LP approach Representative case of the LP approach. An 84-year-old female patient who underwent the LP approach for L3 osteoporotic vertebral collapse. Preoperative posteroanterior (A) and lateral (B) radiographs and sagittal CT myelography (C). Postoperative posteroanterior (D) and lateral (E) radiographs, and CT sagittal image (F). CT: computed tomography, LP approach: lateral-posterior combined approach

The patient was placed in the true lateral position under fluoroscopic guidance and secured with tape. Transthoracic retropleural or retroperitoneal approaches were used, and a dedicated retractor (MaXcess retractor, NuVasive, San Diego, CA, USA) was placed to expose the fractured vertebral body and the upper and lower discs. The surgical approach (transthoracic retropleural vs. retroperitoneal) was selected based on fracture level and patient-specific anatomy on preoperative imaging, including body habitus and the relationship between the fractured vertebra and the rib cage/diaphragm. Generally, fractures around T12-L1 more often required a transthoracic retropleural approach to obtain an appropriate working space, whereas lower levels were typically approached via a retroperitoneal route. The discs above and below the affected vertebral body were removed. After coagulation of the segmental vessels, corpectomy was performed using a large osteotome. The upper and lower cartilaginous endplates were carefully removed using a curette. The vertebral column was reconstructed with an expandable titanium cage featuring a rectangular footplate (X-Core2, NuVasive, San Diego, CA, USA). The rectangular footprint was intended to provide anterior column support and distribute load across the endplates. Bone grafting was performed inside and outside the cage using allogeneic bone or artificial tricalcium phosphate particles, excised vertebrae, and excised rib fragments. After positioning the patient in the prone position, the spinal canal stenosis site was decompressed, and posterior pedicle screw fixation was performed at one to three levels above and below the fractured vertebra. Pedicle screws were inserted either percutaneously or openly, depending on the case (Figure 2).

The number of instrumented levels (one to three levels above and below) and the choice of percutaneous versus open screw insertion were not determined by a predefined protocol. They were selected at the treating surgeon's discretion. Fusion levels were selected based on overall clinical judgment, considering fracture morphology and level, local kyphosis/instability, pedicle integrity, and perceived bone quality. Because some cases used a mixed technique (percutaneous and open screw insertion within the same patient), we did not perform a meaningful subgroup analysis by screw insertion method. Adjacent-level lateral lumbar interbody fusion (e.g., at L4/5) was performed concomitantly in selected cases at the discretion of the treating surgeon when preoperative imaging demonstrated clinically relevant adjacent-segment degeneration judged to require additional interbody support at the time of index surgery.

Posterior-Approach Vertebral Replacement With Rectangular Parallelepiped Cages

**Figure 3:**
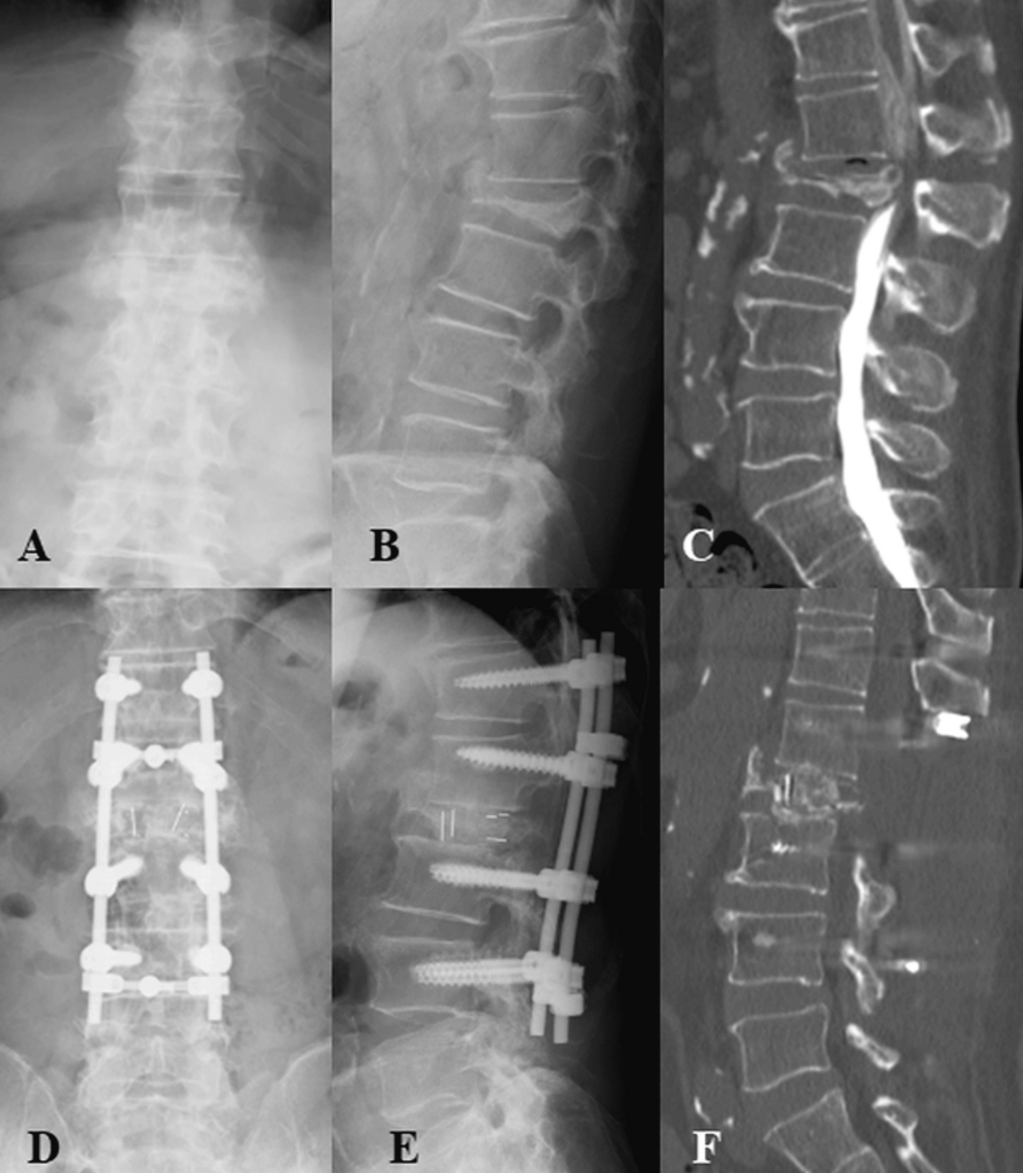
PAVREC Representative case of PAVREC surgery. A 72-year-old male patient who underwent posterior surgery for L2 osteoporotic vertebral collapse. posteroanterior (A) and lateral (B) radiographs and sagittal CT myelography (C). Postoperative posteroanterior (D) and lateral (E) radiographs, and CT sagittal image (F). PAVREC: posterior-approach vertebral replacement with rectangular parallelepiped cages, CT: computed tomography

In posterior-approach vertebral replacement with rectangular parallelepiped cages (PAVREC), the patient was placed prone. The facet joints cranial and caudal to the fractured vertebra were resected to gain access to the intervertebral discs, which were subsequently debrided. Residual lamina and the base of the transverse processes of the fractured vertebra were excised. Using a high-speed diamond burr, the pedicles were removed, facilitating the removal of the fractured vertebral body through a transpedicular approach. The free vertebral fragments with the posterior longitudinal ligament were meticulously detached from the dura mater, ensuring circumferential decompression of the dura. Depending on the severity of osteoporosis, pedicle screws were inserted one to three levels above and below the fractured vertebra. The number of instrumented levels (one to three levels above and below) and supplemental fixation techniques were not determined by a predefined protocol and were selected at the treating surgeon's discretion. These screws were then connected with rods for stabilization. Two large, rectangular cages filled with autologous bone grafts were inserted posteriorly. Compression was applied cranially and caudally to correct the kyphotic deformity while simultaneously shortening the spine. Additional fixation techniques, such as cross-links or sublaminar tape, were used as needed to enhance stability. Finally, an autologous bone graft was placed at the residual facet joint sites cranially and caudally to promote fusion of the facet joints (Figure 3) [17].

Operative techniques and fixation strategies (e.g., construct length, percutaneous versus open screw insertion, and supplemental fixation) were not protocolized across institutions and reflected routine practice and surgeon preference during the study period. Given the multicenter, multi-surgeon design, procedural details and perioperative management were inherently heterogeneous.

Clinical findings, including the reoperation rate, neurological status, ADL function level, radiographic results, and complications at the preoperative, immediate postoperative, and two-year postoperative time points, were retrospectively evaluated. Validated patient-reported outcome measures were not consistently available across institutions in this retrospective dataset and were therefore not analyzed. Additionally, the patients’ clinical records were reviewed for surgical procedures, OVF level, number of fused segments, existing vertebral fractures, operation time, intraoperative blood loss, intraoperative complications, and postoperative osteoporosis pharmacotherapy, including teriparatide use (yes/no) as recorded in the charts. The operating time for anterior and posterior fusion surgery did not include time spent changing the patient's position. The neurological status of each patient was assessed using the Frankel classification. Following the previous report by Hosogane et al., ADL classification was as follows: (1) bedridden, (2) wheelchair, (3) walking while holding on to a wall or creeping, (4) walking with a walker, bilateral canes, or one cane with support from others, (5) walking with a unilateral cane without any support, and (6) walking freely [12].

Radiological findings, such as local kyphosis angle (LKA), implant failure (screw back-out, screw loosening, and rod fracture), and adjacent vertebral fractures after the surgery were evaluated. The LKA was measured as the Cobb angle between the endplate of the vertebra just cranial to the collapsed vertebra and the inferior endplate of the vertebra just caudal to the collapsed vertebra on a lateral-view radiograph preoperatively, immediately postoperatively, and two years after surgery. Screw loosening was defined as a lucent zone around the screw on frontal or lateral radiographic images.

Statistical analysis

Continuous variables were analyzed using the Mann-Whitney U (Wilcoxon rank-sum) test. Categorical variables were compared using Fisher’s exact test; for r×c tables, we additionally report Pearson’s χ² and Cramér’s V as effect-size context, while statistical significance is based on Fisher’s exact p-values. A p-value <0.05 was considered significant in all analyses. Values are presented as medians (interquartile range: 25-75%). Statistical analyses were conducted using EZR software version 1.27 (Saitama Medical Center, Jichi Medical University, Saitama, Japan) [18]. Multivariable adjustment or propensity score analyses were not performed due to the small sample size and incomplete availability of key confounders.

## Results

Comparison of patient demographics

Table 1 summarizes the preoperative baseline characteristics of the patients in the LP and P groups. There were no significant differences in age, sex, illness duration, number of preexisting VFs, fracture-level distribution, number of fused levels, or postoperative follow-up period per patient (Table 1).

**Table 1:** Patients’ demographics characteristics Values are presented as median (interquartile range) or n (%). Continuous/ordinal variables were compared using the Mann-Whitney U (Wilcoxon rank-sum) test. Effect size is reported as probability of superiority (PS; AUC interpretation of the Mann-Whitney U), calculated as U/(n1×n2). Categorical variables were compared using Fisher’s exact test; for r×c tables, Cramér’s V is additionally reported as an effect-size context. VFs: vertebral fractures, LP group: lateral-posterior combined approach group, P group: posterior-only approach group, CI: confidence interval

	LP group	P group	Statistical value/effect size	p-value
No. of cases	22	16		
Age, y	75.0 (72-81)	75.5 (68-78)	PS (AUC) = 0.43	0.468
Sex, male/female	4/18	2/14	Fisher’s exact test	1
Illness duration (m)	5 (3-8)	4 (2-6)	PS (AUC) = 0.41	0.333
No. of preexisting VFs	0 (0-0)	0 (0-1)	PS (AUC) = 0.62	0.145
Fracture level (n, %)			Cramér’s V = 0.40	0.272
T11	0 (0%)	2 (12.5%)		
T12	11 (50.0%)	9 (56.3%)		
L1	4 (18.2%)	4 (25.0%)		
L2	5 (22.7%)	1 (6.3%)		
L3	2 (9.1%)	0 (0%)		
No. of fused level	5 (4-5)	4 (4-5)	PS (AUC) = 0.44	0.530
Post follow-up period (m)	42 (31-51)	42 (31-56)	PS (AUC) = 0.55	0.367

Perioperative and postoperative events

There was no significant difference in the reoperation rate within two years after surgery (LP two cases, 9.1%; P two cases, 12.5%). Regarding reoperation details, the LP group underwent X-Core cage replacement and fixation extension due to adjacent segment disease. In the P group, reoperations included evacuation of an epidural hematoma with laminectomy and additional rod fixation. Postoperative complications in the LP group included pleural injury in one patient, a poorly positioned cage in one patient, and a rod fracture in one patient. In the P group, one patient had an epidural hematoma, and one patient had set screw loosening. There were no significant differences in the frequencies of surgical site infection, screw back out, screw loosening, rod fracture, or adjacent vertebral fracture. Teriparatide was prescribed postoperatively as part of osteoporosis management; however, initiation timing varied and was not consistently documented across institutions (Table 2).

**Table 2:** Perioperative and postoperative events Data are shown as n (%). Categorical variables were analyzed using Fisher’s exact test. Effect sizes are reported as ORs with exact 95% CIs. Teriparatide use indicates postoperative prescription as recorded in the charts; initiation timing varied and was not consistently documented across institutions. VFs: vertebral fractures, LP group: lateral-posterior combined approach group, P group: posterior-only approach group, CI: confidence interval, OR: odds ratio, CI: confidence interval

	LP group (n = 22)	P group (n = 16)	OR (95% CI)	p-value
Teriparatide	8 (36.4%)	5 (31.3%)	1.24 (0.26-6.34)	1.000
Perioperative complication				
Surgical site infection	1 (4.5%)	2 (12.5%)	0.34 (0.005-7.18)	0.562
Screw back out	8 (36.4%)	3 (18.8%)	2.42 (0.44-17.25)	0.296
Screw loosening	10 (45.5%)	11 (68.8%)	0.39 (0.08-1.74)	0.197
Adjacent VFs	5 (22.7%)	4 (25.0%)	0.89 (0.15-5.46)	1.000
Others	3 (13.6%)	2 (12.5%)	-	-
	Rod fracture: 1 case	Set screw loosening: 1 case		
	Pleural injury: 1 case	Epidural hematoma: 1 case		
	Poorly positioned cage: 1 case			
Reoperation for two years	2 (9.1%)	2 (12.5%)	0.71 (0.04-10.84)	1.000

Surgical invasion

**Figure 4:**
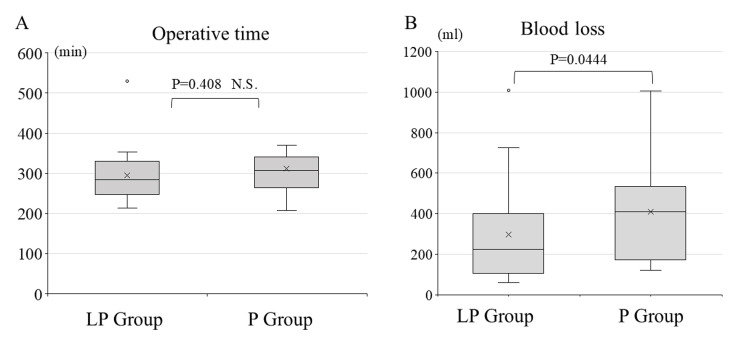
Comparison of operative time (A) and intraoperative bleeding (B) in the LP group and P group Data was analyzed using the Mann-Whitney U test. LP group: lateral-posterior combined approach group, P group: posterior-only approach group, N.S: not significant

The median operation time was 283.5 (250.5-328.5) minutes in the LP group and 306.5 (264.8-333.8) minutes in the P group. The median blood loss was 225.0 (110.0-397.3) mL in the LP group and 410.5 (197.5-520.8) mL in the P group. There was no significant difference in operation time between the two groups, but intraoperative blood loss was significantly less in the LP group (Figure 4).

Correction and correction loss of local kyphosis angle

In the LP group, median LKAs were 28.5° (26.0-32.3°) preoperatively, 3.5° (2.0-12.5°) immediately postoperatively, and 9.5° (4.3-18.5°) at the final follow-up (Table 3).

**Table 3:** Comparison of local kyphosis angle in the LP group and P group, including preoperative, immediately postoperative, at final follow-up, correction angle, and loss of correction Values are presented as median (interquartile range). Between-group comparisons at each time point were performed using the Mann-Whitney U (Wilcoxon rank-sum) test. Effect size is reported as probability of superiority (PS; AUC interpretation of the Mann-Whitney U), calculated as U/(n1×n2). All angles are reported in degrees (°). LP group: lateral-posterior combined approach group, P group: posterior-only approach group

	LP group (n = 22)	P group (n = 16)	PS (AUC)	p-value
Local kyphosis angle				
Preoperative	28.5 (26.0-32.3)	30.5 (21.3-43.8)	0.51	0.953
Immediate postoperative	3.5 (2.0-12.5)	7.0 (2.8-10.8)	0.57	0.476
At final follow-up	9.5 (4.3-18.5)	5.0 (3.0-30.0)	0.41	0.343
Correction	27.0 (21.0-29.8)	22.0 (16.8-27.5)	0.38	0.214
Loss of correction	5.0 (1.3-10.0)	0 (-1-3.3)	0.29	0.028

Immediately after surgery, a median correction of 27.0° (21.0-29.8°) from preoperative levels was observed for kyphosis, with a 5° loss of correction (1.3-10.0°). In the P group, median LKAs were 30.5° (21.3-43.8°) preoperatively, 7.0° (2.8-10.8°) immediately postoperatively, and 5.0° (3.0-30.0°) at the final follow-up. There were no significant differences in preoperative, immediate postoperative, and final follow-up LKAs between the LP and P groups. There was no significant difference in the corrected angle immediately after surgery between the two groups, and the loss of the corrected angle at the final follow-up was significantly greater in the LP group (LP 5° (1.3-10.0°), P 0° (-1-3.3°)).

Neurological deficit (Frankel grade) and activities of daily living

Preoperative and postoperative neurological deficits and ADL grades in the LP and P groups are shown in Table 4. There were no significant differences between the two groups either preoperatively or at two years postoperatively.

**Table 4:** Comparison of Frankel classification and ADL grade Data are shown as n (%). Group comparisons were performed using Fisher’s exact test. Effect size is reported as Cramér’s V. ADL grade: (1) bedridden; (2) wheelchair; (3) walking while holding on to a wall or creeping; (4) walking with a walker, bilateral canes, or one cane with support from others; (5) walking with a unilateral cane without any support; and (6) walking freely. ADL: activities of daily living, LP group: lateral-posterior combined approach group, P group: posterior-only aproach group

	LP group (n = 22)	P group (n = 16)	Cramér’s V	p-value
Frankel classification				
Preoperative			0.31	0.165
C	2 (9.1%)	5 (31.3%)		
D	8 (36.4%)	6 (37.5%)		
E	12 (54.5%)	5 (31.3%)		
2 years postoperative			0.16	0.829
C	1 (4.5%)	0 (0.0%)		
D	4 (18.2%)	4 (25.0%)		
E	17 (77.3%)	12 (75.0%)		
ADL grade				
Preoperative			0.50	0.088
1	1 (4.5%)	6 (37.5%)		
2	3 (13.6%)	4 (25.0%)		
3	1 (4.5%)	0 (0.0%)		
4	7 (31.8%)	2 (12.5%)		
5	7 (31.8%)	3 (18.8%)		
6	3 (13.6%)	1 (6.3%)		
2 years postoperative			0.29	0.653
1	0 (0.0%)	0 (0.0%)		
2	2 (9.1%)	0 (0.0%)		
3	0 (0.0%)	1 (6.3%)		
4	3 (13.6%)	3 (12.5%)		
5	10 (45.5%)	6 (37.5%)		
6	7 (31.8%)	6 (37.5%)		

## Discussion

In this retrospective multicenter study of delayed OVFs with neurological deficit, the LP and P approaches showed no significant between-group differences in reoperation rates, neurological recovery, or ADL at two years. The LP approach was associated with lower intraoperative blood loss, whereas correction loss at final follow-up was greater in the LP group.

This study focused on cases presenting with neurological symptoms secondary to severe vertebral collapse following osteoporotic fractures. Although various surgical strategies have been reported for such cases, including minimally invasive posterior fixation combined with vertebroplasty, concerns remain regarding the long-term durability of the P approach [19,20]. Several reports have indicated that despite initial clinical improvement, the P approach with vertebroplasty is associated with implant-related complications, including screw loosening, rod breakage, and progressive kyphotic deformity during mid- to long-term follow-up [20].

In biomechanical studies, circumferential fixation has been reported to provide greater fixation strength after burst fractures [21]. Therefore, it is considered important to reconstruct the anterior column and perform posterior fixation in surgical treatment for severe vertebral collapse. Restoration of anterior support not only contributes to immediate spinal stability, but it may also help maintain long-term sagittal alignment and prevent mechanical failure, particularly in osteoporotic bone.

Few studies have compared the LP approach with the P approach incorporating osteotomies for OVFs. Previous reports suggest that both approaches achieve satisfactory improvements in LKAs, but the P approach, which includes osteotomies, is more invasive [8]. In contrast, the LP approach using the LIF approach is less invasive and has been widely adopted in Japan [15,22]. In the present study, anterior reconstruction with X-Core demonstrated an advantage in enhancing support, yet the P approach with osteotomies was associated with postoperative clinical improvement. In fact, correction loss at the final follow-up was lower in the P group. However, this finding should be interpreted cautiously because correction maintenance likely depends on multiple factors, including global spinal sagittal alignment, back extensor muscle strength, bone quality, and other patient- and surgeon-related variables. Posterior instrumentation techniques were heterogeneous across cases. Therefore, based on this retrospective analysis, we cannot conclude that either the LP approach or the P approach should be modified.

From a clinical perspective, the LP approach may yield good outcomes without the need for adjunct posterior fixation using three-column osteotomy (3CO), provided that adequate correction and fixation are achieved [19,20]. This approach may be particularly beneficial for elderly patients, since intraoperative blood loss poses a significant risk for severe complications, making minimally invasive strategies preferable. However, because the limited surgical field of the LIF approach used in the LP approach poses a risk of serious organ damage, it is important to have a good understanding of the surrounding anatomical structures to reduce this risk [22]. Meanwhile, 3CO-based posterior fixation, although more invasive, provides more effective correction and may be particularly beneficial in rigid kyphotic deformities with facet joint fusion [23]. The P approach is often used for common OVF procedures and is familiar to most spine surgeons [12]. Previous studies have reported that spinal fusion using 3CO for thoracolumbar OVF resulted in a substantial improvement in local kyphosis but was more invasive than posterior spinal fusion (PSF) alone or vertebroplasty with PSF [8].

Additionally, because the LIF approach is anatomically challenging at the L5 vertebral level, 3CO may serve as an effective alternative for severe L5 vertebral collapse [24]. These findings suggest that both approaches can be viable, and the surgical strategy should be tailored to the patient’s anatomical characteristics, surgical risk profile, and spinal deformity pattern. The P approach may be particularly useful in patients for whom lateral procedures are contraindicated, such as those with comorbidities, anatomical limitations, or poor general condition.

In this comparative analysis of the LP and P approaches for OVFs with neurological impairment, both approaches achieved deformity correction, and no clear between-group differences were observed in perioperative and postoperative event rates. Rates of surgical site infection, screw-related complications, adjacent fractures, and two-year reoperation were low and did not differ between groups; neurologic status and ADL distributions at two years were likewise comparable. Radiographic improvement was observed in both cohorts, and between-group differences in correction were small; a greater loss of correction in the LP approach did not correspond to worse functional outcomes within the study period. These findings indicate that either technique may be considered, with choice guided by fracture morphology, bone quality, and surgeon expertise.

There are several limitations to this study. First, the sample size was small. A priori power analysis was not performed because all eligible patients over a 10-year period were retrospectively included using strict inclusion and exclusion criteria. The study may have been underpowered, increasing the risk of false-negative findings, and it precluded meaningful subgroup analyses. Therefore, our results should be interpreted as exploratory and hypothesis-generating. Second, the indication for surgery was determined subjectively by the surgeon, and the choice of surgical technique was subject to the surgeon's bias. Selection bias is also possible due to strict exclusion criteria and the use of complete-case analysis, which requires a minimum two-year follow-up.

Additionally, inter-surgeon variability and temporal changes in technique over the 2010-2020 study period may have influenced outcomes. We did not perform multivariable adjustment or propensity score analyses due to the small sample size and limited availability of key confounders. Third, bone mineral density (BMD) and detailed osteoporosis-related comorbidities were not analyzed. DXA measurements were not consistently performed or available across institutions during the study period, and therefore, BMD could not be reliably assessed. Unmeasured differences in bone quality and osteoporosis-related factors may have confounded mechanical outcomes, including correction maintenance and implant-related complications. Global sagittal alignment parameters were not systematically assessed, and rehabilitation protocols were not standardized or captured in sufficient detail; both factors may confound functional recovery and radiographic maintenance. Additionally, validated patient-reported outcome measures were not consistently available across institutions and therefore could not be analyzed, limiting the interpretation of patient-centered outcomes. To increase the generalizability of these findings, future research should include larger prospective observational cohorts or randomized controlled trials comparing the surgical techniques evaluated in this study.

## Conclusions

In this multicenter retrospective cohort of osteoporotic thoracolumbar fractures with neurological deficit, the LP approach and the P approach incorporating 3CO were both associated with postoperative neurological and functional improvement at two years. The LP approach was associated with lower intraoperative blood loss, whereas correction loss at two years was lower in the P group. No clear between-group differences were observed in two-year reoperation or perioperative and postoperative event rates. These findings are descriptive and hypothesis-generating, given the retrospective design and limited sample size.
